# Neurosyphilis presenting with unusual hippocampal abnormalities on magnetic resonance imaging and positron emission tomography scans: a case report

**DOI:** 10.1186/1752-1947-6-389

**Published:** 2012-11-21

**Authors:** Taha A Omer, Deirdre E Fitzgerald, Niall Sheehy, Colin P Doherty

**Affiliations:** 1 Department of Neurology, St James’s Hospital, James Street, Dublin, Ireland; 2 Department of General Medicine, St James’s Hospital, James Street, Dublin, Ireland; 3 Department of Radiology, St James’s Hospital, James Street, Dublin, Ireland

## Abstract

**Introduction:**

The incidence of neurosyphilis has declined markedly since the introduction of penicillin therapy. While there are a number of case reports in the literature of neurosyphilis causing focal decreased 18F-fluorodeoxyglucose uptake on positron emission tomography/computed tomography scans, to the best of our knowledge this is the first published report of neurosyphilis presenting with intensely increased 18F-fluorodeoxyglucose uptake in the hippocampus.

**Case presentation:**

A 55-year-old Caucasian man presented to our facility with acute collapse against a background of memory difficulties over the previous six months. The results of his initial physical examination were normal. He scored 24 out of 30 on the Montreal Cognitive Assessment test. A magnetic resonance imaging scan of his brain revealed high T2 signal intensity and atrophy within the right frontal area in addition to high T2 signal intensity in the bilateral mesial temporal areas. Blood and cerebrospinal fluid analysis revealed an active syphilis infection. An 18F-fluorodeoxyglucose positron emission tomography brain scan showed intensely increased 18F-fluorodeoxyglucose uptake limited to the head of the right hippocampus. He responded to penicillin treatment with an improvement in his cognition, which was further reflected in a complete resolution of the findings previously seen on magnetic resonance imaging and 18F-fluorodeoxyglucose positron emission tomography scans.

**Conclusions:**

Diagnosis of neurosyphilis can be difficult, as many patients are either asymptomatic or present with non-specific symptoms such as memory disturbance or seizures. This report highlights the importance of considering neurosyphilis in the differential diagnosis when mesiotemporal changes are seen on magnetic resonance imaging or 18F-fluorodeoxyglucose positron emission tomography scans.

## Introduction

The incidence of neurosyphilis has declined markedly since the introduction of penicillin therapy. Neurosyphilis can occur at any stage of syphilis, although symptomatic early disease is a rare manifestation. Early diagnosis of neurosyphilis and appropriate antibiotic therapy make notable clinical improvement. While there are a number of case reports in the literature of neurosyphilis causing focal decreased 18F-fluorodeoxyglucose (18F-FDG) uptake on positron emission tomography/computed tomography (PET/CT) scan, to the best of our knowledge this is the first published report of neurosyphilis presenting with intensely increased 18F-FDG uptake in the hippocampus.

## Case presentation

**Figure 1:**
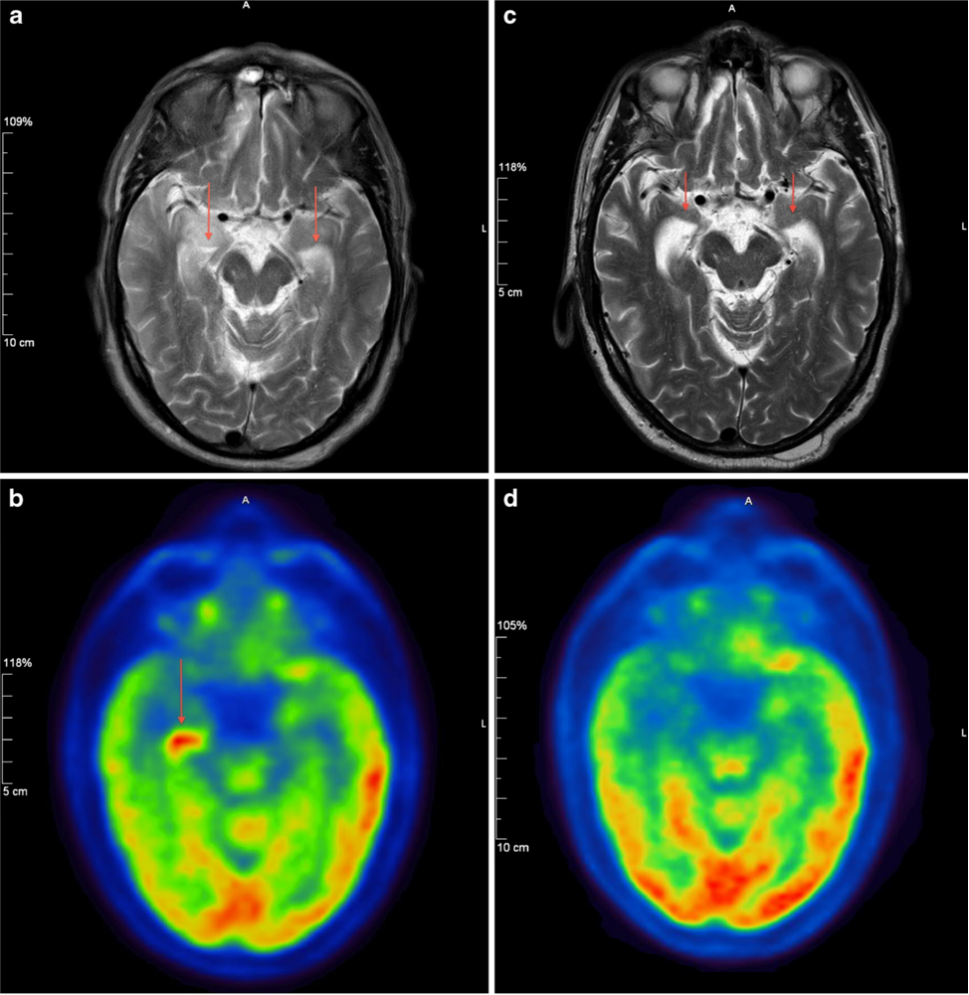
**Brain imaging studies.** (**a**) Pre-treatment brain magnetic resonance imaging scan showing bilateral mesial temporal high T2 signal intensity (arrows). (**b**) Pre-treatment brain positron emission tomography/computed tomography brain scan revealing a focus of intensely increased 18F-fluorodeoxyglucose uptake limited to the head of the right hippocampus (arrow) on a background of globally decreased 18F-fluorodeoxyglucose uptake. **(c)** Post-treatment brain magnetic resonance imaging scan showing marked improvement in the previously identified bilateral hyperintensities, which were replaced by atrophy (arrows). **(d)** Post-treatment positron emission tomography brain scan showing normal 18F-fluorodeoxyglucose uptake in the right hippocampus.

A 55-year-old Caucasian man presented to our facility with acute collapse against a background of memory difficulties over the previous six months. He was a smoker but had no chronic illness and was on no regular medications. Initial physical examination was normal. He scored 24 out of 30 on the Montreal Cognitive Assessment (MOCA): he lost four points on delayed recall, and one point on each of language fluency and orientation domains. Although the collapse was not witnessed, our patient had no memory of the fall and a differential diagnosis of seizure versus syncope was made. The results of hematological, biochemical and cardiovascular investigations were normal. An electroencephalogram (EEG) showed non-specific irregular slowing without epileptiform features. An initial CT scan of the brain showed a right frontal gliosis, probably related to prior trauma. A magnetic resonance imaging (MRI) scan performed on the second day of his admission revealed high T2 signal intensity and atrophy within the right frontal area consistent with the gliosis, but there was also high T2 signal intensity in bilateral mesial temporal areas (Figure 1a). A lumbar puncture was performed and the cerebrospinal fluid (CSF) revealed 16 cells/μL for white blood cells (WBCs) (100 percent mononuclear cells) and elevated protein level (77mg/dl). The CSF glucose level was normal and the results of a Gram stain were negative.

In view of his burgeoning cognitive problems and MRI findings, the possibility of a paraneoplastic (PN) process was considered. A serological screen for PN antibodies was conducted and a CT scan of the thorax, abdomen and pelvis (CT TAP) as well as 18F-FDG PET/CT of the body and brain were obtained. A PET scan of the brain revealed a focus of intensely increased 18-FDG uptake limited to the head of the right hippocampus on a background of globally decreased 18-FDG uptake (Figure 1b).

At this point, at insistence of his family, our patient revealed a history of homosexual contact. Both his blood and CSF were tested for syphilis: his serum serology gave positive results on enzyme immunoassay (EIA), treponemal IgM and *Treponema pallidum* particle agglutination assay (TPPA). His CSF also tested positive for TPPA. Human immunodeficiency virus (HIV) and hepatitis serology results were negative. A diagnosis of neurosyphilis was made and treatment was initiated. He was treated with penicillin G procaine 2.4 million units intra-muscularly daily plus probenecid 500mg four times daily orally, both for 14 days.

Three months later our patient was evaluated at a follow up appointment and was found to have improved cognitive function (scoring 27 out of 30 on repeat MOCA testing). A repeat MRI scan of the brain, performed two months after completing the penicillin treatment course, showed marked improvement in the previously identified bilateral hyperintensities, which were replaced by atrophy (Figure 1c) and a repeat PET brain scan two days after the repeat MRI showed normal 18-FDG uptake in the right hippocampus (Figure 1d).

## Discussion

Neurosyphilis is a slow progressive, destructive infection of the brain and spinal cord caused by the spirochete *T. pallidum*. Its incidence has declined markedly since the introduction of penicillin therapy. It can occur at any stage of syphilis, although symptomatic early neurosyphilis is a rare manifestation [1]. Early diagnosis of neurosyphilis and appropriate antibiotic treatment make notable clinical improvement. However, the clinical diagnosis of neurosyphilis is often difficult because most patients are asymptomatic or present with non-specific symptoms such as memory disturbance or seizures [2,3]. CSF examination is recommended in all patients with untreated syphilis of unknown duration or of duration greater than one year. The diagnosis of neurosyphilis is based on a CSF WBC count of 20 cells/μL or greater, and/or a reactive CSF Venereal Disease Research Laboratory (VDRL) test, and/or a positive CSF intra-thecal *T. pallidum* antibody index [4]. Other CSF abnormalities include elevated protein levels and pleocytosis, which are found in up to 70 percent of patients. In addition, the CSF VDRL result is reactive. Some experts advise lumbar puncture in patients with secondary and early latent syphilis. This is because standard penicillin G benzathine therapy for early syphilis does not achieve treponemicidal levels in the CSF.

Because syphilis serology is not routinely tested for in patients with seizures or amnesia, the possibility of neurosyphilis should be considered in the differential diagnosis when mesiotemporal signal changes are seen on MRI or PET scans. Bilateral mesial temporal lobe hyperintensity may be seen on T2-weighted MRI sequences in a range of acute or chronic conditions. Acute hyperintensity may be seen in herpes simplex encephalitis, paraneoplastic limbic encephalitis, vasculitis and status epilepticus. Chronic hyperintensity along with atrophy is commonly seen in mesial temporal sclerosis and neurodegenerative disorders. In our patient’s case the finding of increased 18F-FDG uptake in the hippocampus on the PET scan gave rise to concerns about ongoing focal seizures in the right hippocampus, however, an EEG was completed within two hours of the PET scan and showed no evidence of seizure. Furthermore, there were no fluctuating cognitive findings or pattern of awareness to suggest seizure activity. Finally, the hypermetabolism outlined exactly the hippocampal head suggesting a structural rather than electric abnormality. 18F-FDG accumulates in inflammatory cells, which presumably explains the increased tracer uptake in our patient.

## Conclusions

While there are a number of case reports in the literature of neurosyphilis causing focal decreased 18F-FDG uptake on PET imaging, to the best of our knowledge, this is the first published case of neurosyphilis presenting with increased 18F-FDG uptake in the hippocampus.

## Consent

Written informed consent was obtained from the patient for publication of this case report and any accompanying images. A copy of the written consent is available for review by the Editor-in-Chief of this journal.

## Competing interests

The authors declare that they have no competing interests.

## Authors’ contributions

CD was the consultant neurologist who cared for our patient at the time of presentation and made the diagnosis. TO was the registrar attached to the neurology unit at the time and is responsible for drafting the case report. NS was the consultant radiologist who reported on the scans. DF was the general medical registrar who evaluated our patient at his first presentation to the hospital. All authors read and approved the final manuscript.
